# Fluorescent Protein-Based Autophagy Biosensors

**DOI:** 10.3390/ma14113019

**Published:** 2021-06-02

**Authors:** Heejung Kim, Jihye Seong

**Affiliations:** 1 Brain Science Institute, Korea Institute of Science and Technology (KIST), Seoul 02792, Korea; yunwjd7@kist.re.kr; 2 Department of Converging Science and Technology, Kyung Hee University, Seoul 02453, Korea

**Keywords:** autophagy, fluorescence imaging, fluorescent protein, biosensors, neurodegenerative diseases

## Abstract

Autophagy is an essential cellular process of self-degradation for dysfunctional or unnecessary cytosolic constituents and organelles. Dysregulation of autophagy is thus involved in various diseases such as neurodegenerative diseases. To investigate the complex process of autophagy, various biochemical, chemical assays, and imaging methods have been developed. Here we introduce various methods to study autophagy, in particular focusing on the review of designs, principles, and limitations of the fluorescent protein (FP)-based autophagy biosensors. Different physicochemical properties of FPs, such as pH-sensitivity, stability, brightness, spectral profile, and fluorescence resonance energy transfer (FRET), are considered to design autophagy biosensors. These FP-based biosensors allow for sensitive detection and real-time monitoring of autophagy progression in live cells with high spatiotemporal resolution. We also discuss future directions utilizing an optobiochemical strategy to investigate the in-depth mechanisms of autophagy. These cutting-edge technologies will further help us to develop the treatment strategies of autophagy-related diseases.

## 1. Introduction

Autophagy is an important cellular process of self-degradation for dysfunctional or unnecessary molecules and organelles, thus dysregulation of autophagy can be involved in various diseases such as neurodegenerative diseases [1,2,3]. To understand complex process of autophagy and the related diseases, various methods have been developed, for example biochemical, chemical, and imaging assays [4,5,6,7,8]. In particular, fluorescent protein (FP)-based autophagy biosensors allow sensitive and selective monitoring of autophagy progression in live cells [9].

After the discovery of green fluorescent protein (GFP) [10], a variety of FPs has been discovered and engineered which have different physicochemical properties, such as excitation/emission spectra, Stokes shift, maturation rate, stability, photo-reactivity and pH-sensitivity [11,12,13,14,15,16]. Advances in fluorescent protein technology and FP-based biosensors enabled the real-time monitoring of cellular and molecular events in live cells with high spatiotemporal resolutions [17,18,19]. Different sensing strategies of FP-based biosensors have been developed [20], in particular biosensors to monitor the progression of autophagy are based on different FP properties such as pH-sensitivity, stability, brightness, spectral profile, and fluorescence resonance energy transfer (FRET) [12,14,21,22,23,24,25,26,27,28,29,30,31,32,33,34].

In this review, we first explain the autophagy types and mechanisms, and discuss how autophagy is involved in neurodegenerative diseases. We then overview different methods to study autophagy and discuss the principles and limitations of these methods. We next focus on the review of the FP-based autophagy biosensors, which are based on autophagy biomarkers tagged with single FP, tandem or triple FPs with different pH-sensitivity, FRET and photoconversion. These FP-based autophagy biosensors can be applied to investigate complex mechanisms of autophagy as well as pathological mechanisms of related diseases such as neurodegenerative diseases.

## 2. Autophagy and Neurodegenerative Diseases 

In this section, we explain three types of autophagy and related molecular mechanisms: macroautophagy, microautophagy and chaperone-mediated autophagy (CMA). Recent progress in autophagy-related diseases has been elaborated well in other fine reviews [5,35,36], thus here we focus on how autophagy is related in particular to neurodegenerative diseases such as Parkinson’s disease, Alzheimer’s disease and Huntington’s disease.

### 2.1. Autophagy Types

**Figure 1:**
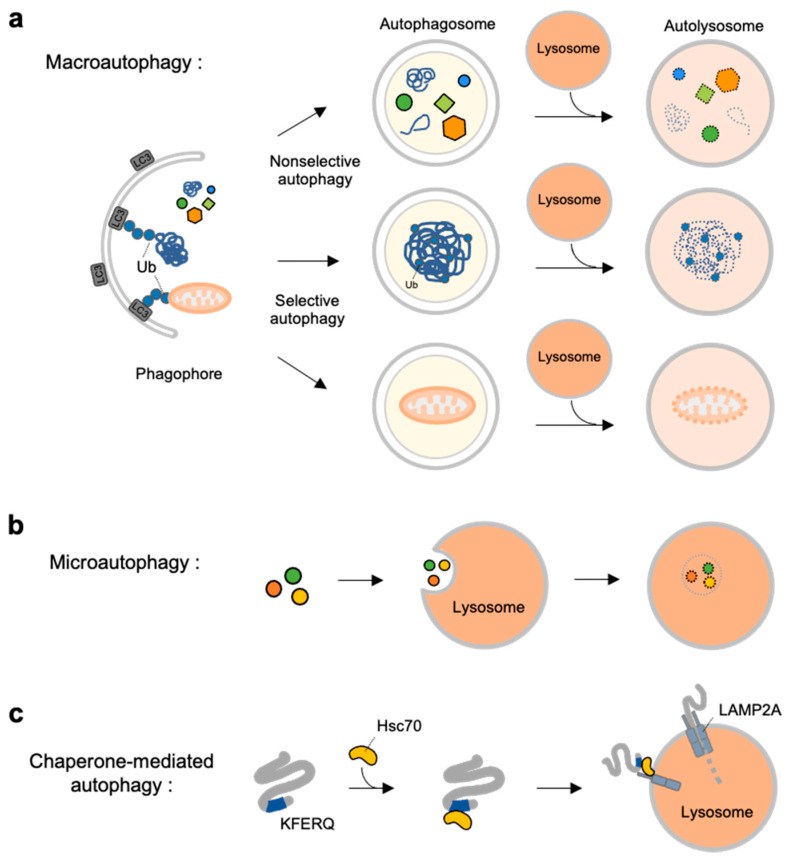
Overview of three types autophagy. (**a**) Macroautophagy is initiated by assembly of double-membrane called phagophore. Non-selective or selective cargoes are enclosed in autophagosomes. Autophagosomes fuse with lysosomes to become autolysosomes. The cargoes within autolysosome are digested by lysosomal enzymes. (**b**) In microautophagy, the cargoes directly engulfed by lysosomal membrane and degraded in the lysosome. (**c**) In chaperone-mediated autophagy (CMA), a selective subset of cytosolic proteins containing a KFERQ-motif is recognized by heat shock cognate protein of 70 kDa (Hsc70). They are delivered to lysosomal membrane by binding to lysosomal-associated membrane protein 2A (LAMP2A). The cargoes are unfolded and translocated inside the lysosome where they are degraded by lysosomal enzymes.

Autophagy is a self-degradation process for dysfunctional or unnecessary cellular components through lysosomal machinery. Depending on the delivery mechanisms to lysosomes and types of cargo, autophagy can be categorized into macroautophagy, microautophagy and chaperone-mediated autophagy (CMA) [37] (Figure 1).

Macroautophagy is initiated by assembly of double-membrane phagophore, and the cargoes are sequestrated within double-membrane vesicles known as autophagosomes [38] (Figure 1a). Autophagosomes then fuse to the lysosome, and the enclosed cargoes are digested by lysosomal enzymes. Macroautophagy can be non-selective or selective: non-selective macroautophagy is a bulk type of autophagy process under starvation for cell survival, and selective macroautophagy is for the degradation of specific substrates, e.g., organelles, aggregated proteins and dysfunctional intracellular components [39]. Selective macroautophagy is named for its special cargoes. For example, mitophagy is selective degradation process of dysfunctional mitochondria, which is often impaired in many diseases such as neurodegenerative diseases, cancer, and metabolic disorder [40]. Other examples for specific macroautophagy include ERphagy, aggrephagy, lipophagy, ribophagy [41].

In contrast to macroautophagy, microautophagy and CMA are the types of autophagy directly occur at lysosomes. In microautophagy, the cargoes are not recruited to the double-membrane autophagosomes but, instead, are directly engulfed by lysosomal membrane [42] (Figure 1b). Finally, chaperone-mediated autophagy is a special autophagy mechanism for a selective subset of cytosolic proteins containing a KFERQ-motif [43] (Figure 1c). The exposed motif on the cargoes can be specifically recognized by heat shock cognate protein of 70 kDa (Hsc70), and delivered to lysosomal membrane via the interaction of the Hsc70 with lysosomal-associated membrane protein 2A (LAMP2A) [44]. The cargoes are then unfolded and translocated inside lysosome where they can finally be degraded by lysosomal enzymes [43,45].

### 2.2. Molecular Mechanisms at Different Stages of Macroautophagy

Among three types of autophagy, the molecular mechanisms of macroautophagy have been most extensively studied [46,47]. Macroautophagy is initiated by AMP-activated protein kinase (AMPK) activation [48] or mechanistic target of rapamycin (mTOR) inhibition [49], which lead to the subsequent activation of the Unc-51 like kinase 1 (ULK1) complex, comprised of ULK1, autophagy-related protein 13 (Atg13), focal adhesion kinase family interacting protein of 200-kDa (FIP200) and Atg101 [50]. The activated ULK1 mediates Atg9 trafficking for the formation of isolation membranes called phagophore [51,52]. In addition, ULK1 activates the Vps34 complex, composed of Vps34, Vps15, Atg14, and Beclin-1, inducing the generation of phosphatidylinositol 3-phosphate (PI3P) [53]. ULK1 also phosphorylates activating molecule in Beclin 1-regulated autophagy protein 1 (AMBRA1), promoting the translocation of the Vps34 complex to the phagophore [50]. The PI3P-binding proteins, WD-repeat protein interacting with phosphoinositide 1 (WIPI1) and WIPI2, are then recruited at the phagophore and catalyze the ubiquitination-like reactions for the elongation of phagophore [54]. 

The closure of phagophore is facilitated by the Atg12- Atg5-Atg16L complex, which is conjugated by the Atg7 and Atg10 [55]. This complex promotes the lipidation to Atg8, also called microtubule-associated protein light chain 3 (LC3). Before this step, pro-LC3 is processed by Atg4 to LC3-I which exposes a C-terminal glycine residue. Phosphatidylethanolamine (PE) is then attached to the glycine residue of LC3-I converting it to LC3-II [56]. LC3-II is incorporated at the membrane of autophagosomes and recruits the cargoes bound to autophagy receptors such as SQSTM1/p62, optineurin, NBR1 and NDP52 [57,58,59,60]. LC3-II can be present at both inner and outer surface of the newly formed autophagosomes, then the one at the outer surface is eliminated by Atg4 activity [61]. Autophagosomes fuse to lysosomes generating autolysosomes, and this process is mediated by Rab GTPases, soluble N-Ethylmaleimide-Sensitive Factor Attachment Protein Receptor (SNARE) and tethering factors [62,63]. Autolysosomes are further matured, and finally the recruited cargoes inside autolysosomes are degraded by the lysosomal enzymes [3,64]. Therefore, the stages of macroautophagy can be divided by (1) phagophore formation and elongation, (2) autophagosome formation and cargo enclosure, (3) autophagosome-lysosome fusion and autolysosome formation, and (4) lysosomal degradation. As described above, these stages of macroautophagy are tightly regulated by complex molecular mechanisms with spatial and temporal manners.

### 2.3. Autophagy Dysfunctions in Neurodegenerative Diseases

Autophagy is crucial for the removal of damaged organelles or aggregation-prone misfolded proteins. Thus, autophagic dysfunction is often found in neurodegenerative diseases, which are featured by the accumulation of toxic aggregates or damaged organelles [65]. For example, Huntington’s disease (HD) is caused by an expansion of the CAG trinucleotide repeat encoding a polyglutamine (polyQ) tract in the N-terminal of Huntingtin (Htt) protein [66]. More than 36 polyQ repeats in HTT cause the formation of toxic Htt aggregates, which are suggested to be cleared by autophagy. However, decreased ability of autophagic vesicles to recognize cytosolic cargos and reduced autophagosomes have been reported in HD [67]. The normal transport of autophagosomes for the fusion with lysosomes is also defective in HD neurons [68]. As a compensatory mechanism, CMA activity is shown to be upregulated in HD [69]. 

Alzheimer’s disease (AD) is featured by senile plaques composed of amyloid-beta (Aβ) aggregates [70]. Accumulation of autophagosomes and autolysosomes are often observed in AD, suggesting that autophagic flux is impaired [71]. Presenilin 1 (PS-1) mutations, which are known to produce Aβ, are also suggested to impair the acidification of lysosomes thus contributing to dysfunctions in autophagic flux [72]. The released Aβ can exist as monomeric or oligomeric forms and, interestingly, it has been suggested that different Aβ forms have differential effects on autophagy: monomeric Aβ hampers the formation of Bcl-2-Beclin-1 complex and inhibits lysosomal degradation for normal autophagic flux, leading to intracellular accumulation of autophagosomes, while Aβ oligomers facilitate the production of the Bcl-2-Beclin-1 complex thus favoring apoptosis [73]. In addition to Aβ, neurofibrillary tangles from Tau aggregation is another hallmark of AD [74]. Wild-type Tau is degraded by endosomal microautophagy or CMA, but the hyperphosphorylated Tau is subjected to macroautophagy, which is often failed for Tau aggregates [75,76]. 

Parkinson’s disease (PD) is characterized by neuronal inclusions composed of α-synuclein aggregates, which cause the death of dopaminergic neurons in brain [77]. Normal α-synuclein is primarily degraded by CMA which is impaired in PD [78,79]. Accumulated α-synucleins also dysregulate macroautophagy by inhibition of Rab1 GTPase, which results in the mislocalization of Atg9 [80] thus the failure of phagophore formation [81]. In addition to the dysregulation at this initiation step of macroautophagy, α-synuclein aggregates can also impair the maturation of autophagosome and its fusion with lysosome [82]. For example, neurons with α-synuclein aggregates showed a significantly decreased mobility of autophagosomes toward both retrograde and anterograde directions [83]. This impaired mobility inhibits the fusion process with lysosomes thus resulting in the inhibition of autophagic degradation. Finally, defects in the selective degradation of damaged mitochondria are observed in PD [84]. This failure in mitophagy is related to the mutations in PINK1 and Parkin [85], which are major elements for ubiquitin assembly of damaged mitochondria [86].

As described above, dysfunctions at different stages of all types of autophagy, i.e., CMA, macroautophagy and mitophagy, are reported in various neurodegenerative diseases. Thus, it is crucial to correctly understand the molecular mechanisms of autophagy progression, in particular for the investigation of the strategies to overcome these autophagy-related diseases.

## 3. Methods for the Detection of Autophagy

To investigate the molecular mechanisms of autophagy and pathology of the related diseases, it is important to sensitively and accurately monitor the progression of autophagy. Various methods have been applied for the detection of autophagy, for example electron microscopy, radioactive isotope-based assay, biochemical assay, and fluorescence imaging [87,88,89]. In this section, we discuss the principles and limitations of these methods.

### 3.1. Electron Microscopy

Autophagy was first discovered in 1950s from direct observation of autophagic vacuoles containing mitochondria by transmission electron microscopy (TEM) [90]. TEM is an imaging technique to observe the morphology and structure of the specimen by transmission of a beam of electrons [91]. Currently, biochemical assays and fluorescence imaging methods are most widely used to study autophagy, but TEM was the only method to detect autophagy before biochemical assays are developed and specific protein markers for autophagy were discovered in 1990s [91]. TEM still provides indispensable information of ultrastructural details for autophagy, however it is not an optimal method to identify the stages of autophagy or quantify autophagic flux [92], and sample preparation for TEM is more complex than other available methods.

### 3.2. Autophagic Protein Degradation Assay

For the quantification of autophagic flux, the stable isotope labeling by amino acid in cell culture (SILAC) technique have been applied to measure the degradation rate of long-lived proteins by mass spectrometry [93]. In contrast to short-lived proteins, which are cleared by proteasomes, long-lived proteins are thought to be degraded by autophagic pathways. Thus, after incorporation of ^15^N/^13^C-labeled amino acids in the proteins, the radioactive isotope from the long-lived proteins can be measured over time [94,95]. The SILAC assay is suitable for evaluating general degradation rates of the autophagic proteins which represent autophagic flux.

To avoid the use of radioactive materials, autophagic sequestration of an endogenous protein lactate dehydrogenase (LDH) was also measured for the quantification of autophagic flux [96]. LDH is an abundant cytosolic enzyme that is non-selectively wrapped into autophagosomes, thus the sequestration of LDH in autophagic vacuoles and the reduced LDH activity in the cytosol can report the degree of autophagy. These protein degradation assays are useful to inform the general degree of autophagic flux, however, it is limited to identifying different stages of autophagy or monitoring specific molecular events of autophagy mechanisms.

### 3.3. Biochemical Assay

After the discovery of key biomarkers for autophagy [97,98,99], Western blotting of LC3-II and p62 became a traditional method to study autophagy. As described in the Section 2.2, the lipidation on LC3-I and the formation of LC3-II are critical molecular events for autophagy initiation [64]. The LC3-II is present at the membrane of autolysosomes from the early stage of autophagy until degraded in autolysosomes. Thus, the stages of autophagy can be tracked by Western blotting of LC3-I and LC3-II, which are distinguished by the bands at approximately 16–18 kDa and 14–16 kDa on sodium dodecyl sulfate polyacrylamide gel electrophoresis (SDS-PAGE) gels [100,101]. During the autophagy progression, the conversion from LC3-I to LC3-II band and the subsequent disappearance of LC3-II band can be observed by Western blotting [102].

Blockage of autophagic flux and lysosomal degradation can be suspected by the continuous accumulation of LC3-II band of the Western samples. However, that can be also due to excessive induction of autophagy. To distinguish between excessive induction of autophagy and blockage of autophagic flux, the LC3-II bands can be further compared without and with lysosomal inhibitors such as chloroquine and bafilomycin A1 [103,104]. If the LC3-II accumulation of the samples is further increased by lysosomal inhibitors, it can be due to the excessive induction of autophagy, while the case of blockage of autophagic flux may not be affected by the treatment of inhibitors.

Other autophagy biomarkers are also accessed by Western blotting to demonstrate the progression of autophagy. The most common example is SQSTM1/p62, a major receptor for various ubiquitinated cargoes that brings them to LC3-II-positive autophagosomes [60]. As p62 is degraded together with the cargoes in autolysosomes, the decreased band of p62 can represent the autophagy flux. Therefore, the Western blotting of the time-lapse changes of LC3-II and/or p62 allow for the detection of autophagy progression. However, the preparation of cell lysates is required for Western blotting assay, thus it cannot provide real-time spatiotemporal information of autophagy progression in live cells.

### 3.4. Chemical Probes

A fluorescent chemical probe named Seoul-Fluor 44 (SF44) was developed [105] and applied for the monitoring of autophagy flux in live cells [106]. Because this indolizine-based chemical moiety becomes bright in the hydrophobic lipid droplets (LDs), which is primarily degraded by autophagy, the number and intensity of SF44-containing LDs are inversely correlated with autophagy flux. Thus, the degree of autophagy in live cells can be quantified by monitoring SF44-positive LDs with a fluorescence microscope. In particular, this method does not require genetic perturbation, washing or fixation steps, and thus can be applied to a high throughput screening system for autophagy modulators.

In addition to autophagosomes, the autolysosomes were monitored by a pH-detecting plasmon Raman probe using surface-enhanced Raman scattering imaging [107]. This sensitive pH-detecting probe consists of gold nanostars as Raman enhanced substrate, 4-mercaptopyridine as Raman reporter molecules, and bovine serum albumin as protective molecules. The probe allows for the sensitive monitoring of pH changes in lysosomes during autophagy. However, these chemical probes are not optimal for the investigation of molecular mechanisms during autophagy progression, which requires the specific targeting of the probes to autophagy biomarkers.

### 3.5. Fluorescent Protein (FP)-Based Biosensors

Fluorescent proteins are genetically encodable thus can be specifically fused to proteins of interests, allowing for the visualization of their distribution and motions in live cells [17,108]. FP-based biosensors have been developed based on different physicochemical properties of FPs and applied to investigate various molecular and cellular events in live cells [20]. For the monitoring of autophagy progression, FP-based autophagy flux biosensors are designed utilizing different pH-sensitivities of tandem or triple FPs which allow for the detection of pH changes in autophagic vesicles during autophagy progression [109,110,111,112,113,114]. The FP-based autophagy biosensors are specifically tagged with autophagy biomarkers such as LC3 or KFERQ-motif [110,112,113,115], and they can be further targeted to particular subcellular regions, for example mitochondria [116]. These unique features of FP-based autophagy biosensors enabled the sensitive and selective monitoring of the real-time progression of autophagic stages in live cells.

## 4. FP-Based Biosensors for the Monitoring of Autophagy Progression

In the previous section, we have overviewed various methods to detect or monitor autophagy. In this section, we further focus on the FP-based autophagy biosensors which can monitor the progression of autophagy in live cells. These biosensors are dependent on different pH-sensitivities of tandem or triple FPs. When we choose pH-sensitive FPs for the detection of pH changes in autophagic vesicles, two important factors need to be considered. First, pKa values of the FPs need to be in the physiological pH ranges (between pH 5.5 and 7.5) to detect the changes in fluorescent intensity of autophagic vesicles. Second, the fold changes of fluorescent intensity in this pH range need to be large to be appropriate for the detection of pH changes in autophagic vesicles.

The first engineered pH-sensitive FP is pHluourin (pKa = 7.1), which markedly decreases its green fluorescence below pH 7 [117]. Its improved version, superecliptic pHluourin (SEP, pKa = 7.2), is most widely used as it shows dramatic change of fluorescent intensity (about 50-fold) in the pH ranges between pH 5.5 and pH 7.5 [15]. For the red pH-sensitive FP, which can be used in parallel with green probes, pHTomato (pKa = 7.8) was first developed from monomeric red fluorescent protein (mRFP) and mStrawberry [14]. The improved red pH-sensitive FP, pHuji (pKa = 7.7), was developed from mApple, and it shows 22-fold change of fluorescent intensity between pH 5.5 and pH 7.5 [16]. pHmScarlet (pKa = 7.4), which is recently engineered from mScarlet-I, shows a similar pH-dependent response (26-fold change) but improved brightness and photostability [26]. As the other color FP, pHoran4 (pKa = 7.5) is an orange pH-sensitive FP (17-fold change), engineered from mOrange [16]. In contrast to the FPs which lose the fluorescence signals at acidic pH conditions, pHRed (pKa = 6.6) derived from mKeima increases its fluorescence about 10-fold (ex: 585 nm, em: 610 nm) with decreased emission intensity by 440 nm excitation (ex: 440 nm, em: 610 nm) [33]. Thus, the ratiometric imaging of pHRed by dual excitation can be applied for the visualization of acidic cellular environments [33].

The physicochemical properties of different FPs are summarized in Table 1. We now review the designs, principles and limitations of currently available FP-based autophagy biosensors.

### 4.1. GFP-LC3

**Figure 2:**
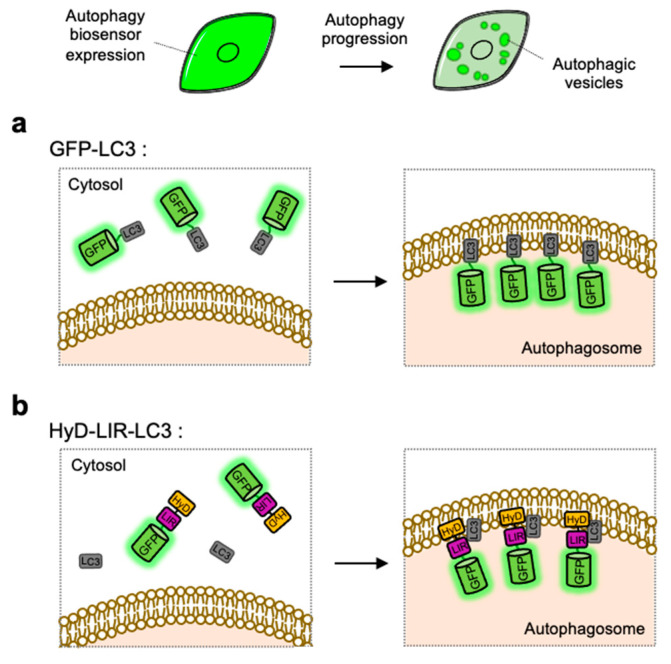
Detection of autophagic vesicles by single FP-tagged light chain 3 (LC3) or LC3 interacting region (LIR) domain. When autophagy is induced, single green fluorescent protein (GFP)-tagged LC3 or LIR domains can change its distribution from whole cell area to autophagic vesicles displaying as bright green puncta. (**a**) Scheme of the GFP-LC3 before and after autophagy induction. (**b**) Scheme of the hydrophobic domain (HyD)-LIR-LC3 before and after autophagy induction. LIR: LC3-interacting region, HyD: hydrophobic domain.

The simple strategy of FP-based biosensor to visualize autophagy progression in live cells is utilizing GFP-tagged LC3, which can directly monitor the formation of LC3-containing autophagosomes [100] (Figure 2). As described in the Section 2.2 and Section 3.3, LC3-II can be a representative autophagy marker presents at autophagic vesicles [118]. GFP-tagged LC3 expressed in cells are observed as fluorescent puncta or ring-shaped structures, indicating the existence of autophagosomes or autolysosomes. The number of GFP-LC3 puncta can be used as an indicator of autophagy induction [119,120]. LC3 tagged with various colors of FPs, for example EGFP, YFP, CFP, RFP, mCherry and HcRed, have been used to monitor autophagosomes in live cells [121,122,123,124,125,126] (Figure 2a).

However, in a simple observation of LC3-positive vesicles it is difficult to identify the stages of autophagy progression, thus additional makers for lysosome such as LysoTracker are required to distinguish between autophagosome and autolysosome [89]. In addition, as explained in Section 3, lysosomal inhibitors need to be further applied when we test whether the accumulation of LC3-positive puncta is derived from excessive induction of autophagy or blockage of autophagic flux.

### 4.2. HyD-LIR-GFP

LC3-positive autophagosomes can be directly monitored by GFP-tagged LC3, however the observed puncta are not from endogenous LC3 but from overexpressed exogenous LC3 [127]. For the visualization of endogenous LC3, HyD-LIR-GFP was developed which is composed of a short hydrophobic domain (HyD) for membrane targeting, a LC3 interacting region (LIR) motif, and GFP [128] (Figure 2b). After, the LIR motifs from 34 LC3-interacting proteins were screened, it was shown that HyD-LIR(Fy)-GFP, containing the LIR motif from FYCO1, allows the specific visualization of LC3-positive autophagosomes. Therefore, HyD-LIR-GFP can be utilized as autophagy biosensors which directly label endogenous LC3-positive autophagosomes.

While HyD-LIR-GFP can avoid the overexpression of exogenous LC3 which may cause unwanted effects, this biosensor also can cause the overexpression of LIR motif which may compete with endogenous LIR-containing proteins. Thus it is generally important to optimize the expression level of the exogenous biosensors. In addition, as in the case of LC3-GFP, it is still difficult to identify exact stages of autophagy by the detection of autophagic vesicles with HyD-LIR-GFP.

### 4.3. RFP-GFP-LC3

**Figure 3:**
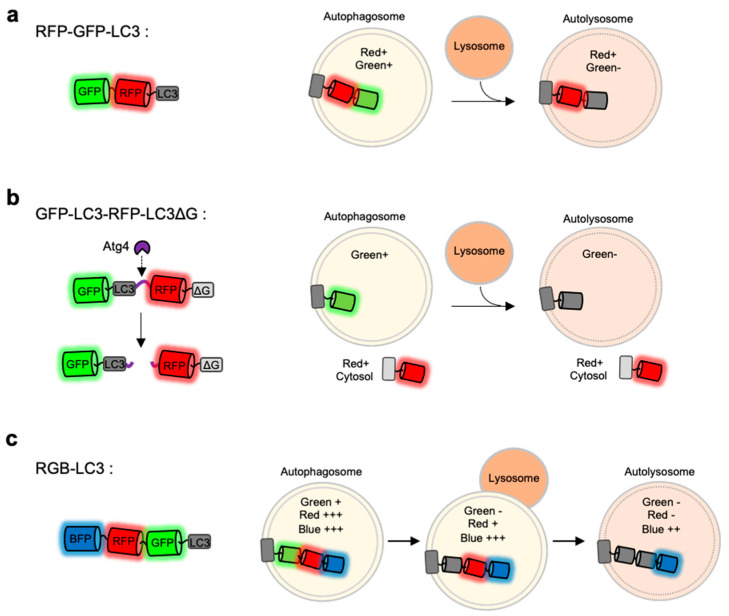
Monitoring of autophagy progression by tandem or triple FPs with different pH-sensitivity (**a**) RFP-GFP-LC3 is a tandem FP-tagged LC3 sensor consisting a pH-sensitive GFP and a pH-stable RFP. Both green and red signals are expected in autophagosomes, while green signal disappears in autolysosomes. (**b**) GFP-LC3-RFP-LC3∆G is composed of GFP-LC3 and RFP-LC3∆G linked by a substrate for an autophagy initiating protease Atg4. When autophagy is induced, the activated Atg cleaves the substrate, generating GFP-LC3 and RFP-LC3∆G. The released GFP-LC3 can be localized at autophagic vesicles, while the RFP-LC3∆G remains in the cytosol serving as an internal control for GFP-LC3. (**c**) Red-green-blue-LC3 (RGB-LC3) is composed of three colors of FPs with different pH-sensitivity: a highly pH-sensitive superecliptic pHluourin (SEP, pKa = 7.2), a pH-stable mTagBFP2 (pKa = 2.7), and mApple (pKa = 6.5) with intermediate pKa and pH-sensitivity. The RGB-LC3 sensor allows for the monitoring of different stages of autophagy.

As discussed above, the monitoring of LC3-positive autophagosomes by single FP-tagged autophagy biosensors is not sufficient to report different stages of autophagy. During the progression of autophagy, the pH inside autophagic vesicles changes to be more acidic, pH-sensitive FPs are applied to distinguish between autophagosomes and autolysosomes [111,114,129]. In particular, LC3 was fused to tandem FPs with different pH-sensitivity and spectral profiles [109,111,114,129] (Figure 3a).

For example, mRFP-GFP-LC3 includes a pH-sensitive EGFP (pKa = 6.0), a relatively pH-stable mRFP (pKa = 4.5) [111]. When the LC3-positive autophagic vesicles become acidic by the fusion of lysosomes, the GFP signal decreases while mRFP signal can be remained. Thus, autophagy progression can be predicted by detecting green and red signals at the LC3-positive vesicles. Similarly, mCherry-EGFP-LC3 shows the pH-sensitive green signal from EGFP (pKa = 6.0) and relatively stable red signal from mCherry (pKa = 4.5) [60]. However, EGFP in these sensors still shows a weak fluorescence even at acidic autolysosomes [114,130], thus green^+^ red^+^ autophagic vesicles can be both autophagosomes and autolysosomes. Therefore, careful quantification is required for the accurate analysis of autophagy status.

To overcome this limitation, the mTagRFP-mWasabi-LC3 was developed [114]. The pKa of mWasabi is 6.5 [131], thus compared to EGFP, its fluorescence decreases at acidic environments more sensitively. In addition, mTagRFP (pKa = 3.8) is very stable in acidic pH [17,132]. However, the green signal from mWasabi was still detected at autolysosomes, thus green^+^ red^+^ and green^−^ red^+^ signals cannot still represent autophagosome and autolysosomes, respectively.

Next, mKate-SEP-LC3 was developed, which contains a highly pH-sensitive SEP (pKa = 7.2) and a relatively stable mKate (pKa = 5.4) [113]. As the pKa of SEP is around 7 and the intensity change is around 50-fold between pH 5.5 and pH 7.5 [117], the autolysosomes can be represented by green^−^ red^+^ signals of mKate-SEP-LC3. However, the highly pH-sensitive SEP signal can be also lost even in the autophagosomes, thus green^−^ red^+^ vesicles may be detected from autophagosomes as well as autolysosomes.

### 4.4. Red, Green and Blue (RGB)-LC3

The FP-based autophagy biosensors composed of LC3 and tandem FPs are not sufficient to identify different status of autophagy progression. Thus the autophagy flux biosensor Red-Green-Blue-LC3 (RGB-LC3) was recently developed, which is composed of three colors of FPs with different pH-sensitivity [110] (Figure 3c). For the FPs in the RGB-LC3, SEP (pKa = 7.2) was selected as a highly pH-sensitive green FP, and mTagBFP2 (pKa = 2.7) was chosen for a pH-stable blue FP. Additionally, mApple (pKa = 6.5) [133], a red FP whose pKa value and the pH-sensitivity are between the ones from SEP and mTagBFP2, was selected to complete RGB-LC3. After the induction of autophagy in the cells expressing RGB-LC3, the formation of autophagosome can be detected by the disappearance of the SEP signal. During the progression of autophagy, the red signal from mApple gradually decreases while mTagBFP2 signal is very stable. Thus the intensity ratios of mApple/BFP2 at the LC-positive autophagic vesicles can display the pH changes in the ranges of the entire autophagy progression, i.e., phagophore, autophagosome, autophagosome-lysosome fusion, and autolysosomes. Therefore, the RGB-LC3 allows for the monitoring of different stages of autophagy.

### 4.5. GFP-LC3-RFP-LC3∆G

GFP-LC3-RFP-LC3∆G is composed of GFP-LC3 and RFP-LC3∆G linked by a substrate for the autophagy initiating protease Atg4 [109] (Figure 3b). When autophagy is induced, Atg4 can cleave the biosensor into GFP-LC3 and RFP-LC3∆G. The released GFP-LC3 can be conjugated to PE and localize to the autophagosomes. Conversely, the RFP-LC3∆G does not contain the C-terminal glycine, which is critical for the PE conjugation, thus remains in the cytosol serving as an internal control for GFP-LC3. Therefore, green puncta of the GFP-LC3 part can visualize the formation and degradation of autophagic vesicles during autophagy, while red signal from the RFP-LC3∆G part is constant in the cytosol. Thus, the GFP/RFP signal ratio can represent the status of autophagy progression. This approach was also successfully applied for high-throughput detection of the level of autophagic flux in vivo as well as in cells.

### 4.6. Fluorescence Resonance Energy Transfer (FRET)-Based Autophagy Sensor

pHlameleons are pH sensors based on fluorescence resonance energy transfer (FRET) between a pH-sensitive FP and a pH-stable FP [134]. For example, a pH-sensitive yellow EYFP (pKa = 6.9) and a pH-stable cyan ECFP (pKa = 4.8) can be an acceptor and a donor for the FRET pair of pHlameleons [135,136,137]. This principle was applied to Cy11.5, a chimeric protein composed of tightly concatenated CFP and YFP thus has a highly efficient orientation for FRET [138]. In the pH ranges of pH 5 and pH 9, the emission of the pH-sensitive EYFP decreases at acidic environment thus resulting in the decreased FRET between ECFP and EYFP. pHlameleons were further improved by replacing ECFP with a more stable cyan FP

Variant mTurquoise2 (pKa = 3.1) [12,135]. This FRET-based pH sensor with mTurquoise2 and EYFP was named as pH-Lemon, and further fused to or other organelles to visualize the pH levels of different intracellular vesicles [139]. The LC3-tagged pH-Lemon confirmed the acidification of LC3-positive autophagic vesicles in the subcellular levels. Further validation is required for the application to an autophagy flux biosensor.

### 4.7. Mitophagy Sensor

The FRET-based mitophagy biosensor was recently developed to monitor selective macroautophagy for damaged mitochondria [140]. As a donor for the FRET biosensor, a very stable cyan FP named TOLLES (TOLerance of Lysosomal EnvironmentS) was applied, which is originally from *Anthozoans* and optimized to be resistant to lysosomal degradation as well as acidic pH (pKa < 3) [141]. This stable cyan FP TOLLES was paired with YPet, a yellow FP which is irreversibly denatured and degraded in the lysosomes, and this FRET-based autophagy flux sensor was named the SRAI (signal-retaining autophagy indicator) [140,141]. The SRAI shows strong FRET between TOLLES and YPet in neural cytosol. While TOLLES is stable during the autophagy process, the YPet signal gradually decreases and completely disappears as degraded in the lysosomes. Thus, the autophagy progression can be monitored by the FRET changes between TOLLES and YPet in the SRAI.

**Figure 4:**
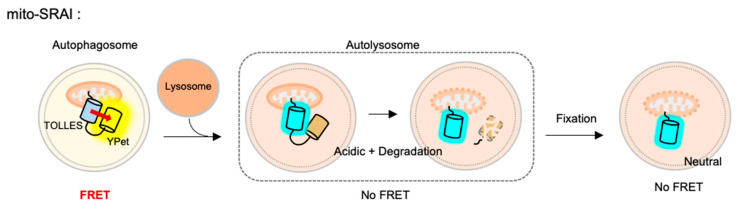
The fluorescence resonance energy transfer (FRET)-based mitophagy biosensor.Mito-SRAI (signal-retaining autophagy indicator) consists a sable cyan FP TOLLES (TOLerance of Lysosomal EnvironmentS) and a yellow FP YPet, as a FRET pair, and strict targeting sequences to mitochondria. Because TOLLES is stable to pH changes and lysosomal degradation, this FRET-based mitophagy sensor allows for accurate monitoring of mitophagy process in live and fixed cells.

For the detection of mitophagy, the SRAI was strictly targeted to mitochondria, through a tandem repeat of the COX VIII presequence [142] and the additional C-terminal degrons CL1 [143] and PEST [144]. This FRET-based mitophagy sensor was named mito-SRAI, and it allows for the monitoring of the lysosomal degradation status by mitophagy in cells and also in vivo [140] (Figure 4). In particular, the signal of a stable cyan TOLLES is fully retained in the fixed cells, mito-SRAI can be successfully applied to large-scale high-throughput screening of a selective mitophagy inducer. Furthermore, mito-SRAI can detect the mitophagy from the fixed brain tissues in a mouse model of Parkinson’s disease.

While other FRET-based autophagy biosensors were focused on different pH-sensitivity of tandem FPs, the SRAI further considered the degradation rates of these FPs in the lysosomes. In particular, if the pH-stable donor FP is easily degraded in the lysosomes, the FRET signals may not be correctly interpreted for the stages of autophagy progression. Thus the discovery of TOLLES, a stable cyan FP resistant to lysosomal degradation as well as acidic pH, is important for the development of the FRET-based autophagy biosensor. Further targeting of the SRAI to other organelles will be useful for the monitoring of selective autophagy.

### 4.8. Chaperone-Mediated Autophagy Sensor

**Figure 5:**
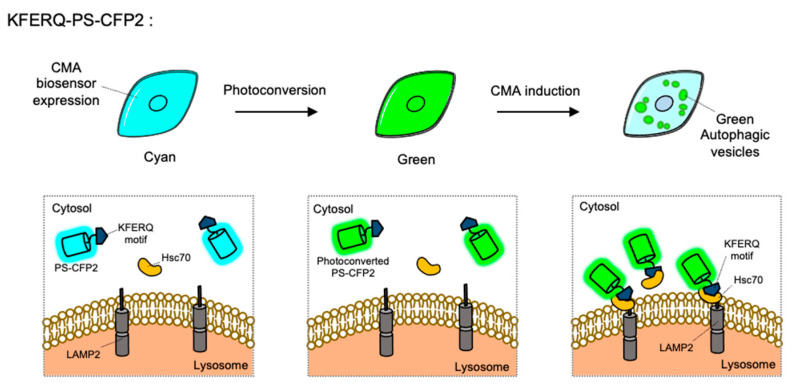
The chaperone-mediated autophagy (CMA) biosensor. The CMA biosensor is composed of the KFERQ-motif and PS-CFP2, a photoconvertible cyan FP. Before photostimulation, the KFERQ-PS-CFP2 is observed as cyan color in whole cell area (**left**). Upon illumination, PS-CFP2 in the CMA biosensor changes its color to green (**middle**). After the induction of CMA, the KFERQ-motif in the CMA biosensor is recognized by Hsc70, which can deliver it to LAMP2 at lysosomes. Thus CMA process can be visualized by green puncta (**right**).

CMA is a special type of autophagy for the proteins containing KFERQ motif. When the KFERQ-motif is exposed from the proteins, the motif is recognized by the cytosolic chaperone Hsc70, and they can be conveyed to the lysosome by interaction of Hsc70 and LAMP2 [43]. For the FP-based CMA biosensor, KFERQ-PS-CFP2 was developed by the fusion of the KFERQ-containing sequences with a photoconvertible cyan FP (PS-CFP2) [115] (Figure 5). In the default state, PS-CFP is a cyan FP (ex: 405 nm, em: 468 nm), but upon photoactivation with 405 nm, PS-CFP is irreversibly converted to a green FP (ex: 490 nm, em: 511 nm) [145]. Thus, a photoconverted population of KFERQ-PS-CFP2 can be selectively monitored by green puncta near the lysosomal membranes. Newly synthesized KFERQ-PS-CFP2 after the illumination will be cyan in the cytosol, thus it can provide a good contrast to green puncta which reporting the CMA. Similarly, a photoactivable mCherry-fused CMA reporter, KFERQ-PA-mCherry, has also been generated as a red version of CMA sensor.

## 5. Summary and Perspectives

Autophagy plays crucial roles in the degradation of misfolded proteins, aggregates, and damaged organelles, thus autophagic dysfunction is closely related to neurodegenerative diseases, which are featured by accumulations of toxic aggregates or damaged organelles. In this review, we discussed biochemical, chemical assays, and imaging methods for the investigation of autophagy and the related diseases. In particular, we introduced the principles and limitations of various FP-based autophagy biosensors which can sensitively visualize the progression of autophagy in live cells. For example, simple tagging of FP to an autophagy marker LC3 allows for monitoring of autophagosomes. The LC3-tagged tandem or triple FPs of different colors and pH-sensitivity can provide further information of autophagy flux by detecting the pH changes inside autophagic vesicles. The FRET-based autophagy sensor with specific mitochondria targeting sequence was applied for the monitoring of mitophagy, and CMA sensors were developed by fusion of KFERQ-motif and photoconvertible FP. These autophagy biosensors and their applications to study neurodegenerative diseases are summarized in Table 2.

Compared to other methods to monitor autophagy such as TEM, biochemical assays and chemical probes, FP-based biosensors allow for more sensitive and selective real-time monitoring of autophagy progression in live cells. For example, these genetically encodable FP biosensors can be specifically tagged with autophagy biomarkers such as LC3 or KFERQ-motif, thus specific types of autophagy can be visualized in live cells with high spatiotemporal resolutions. Second, FP-based autophagy biosensors can be selectively targeted to particular subcellular regions, for example mitochondria, which allows the detection of selective macroautophagy, e.g., mitophagy. Furthermore, pH-sensitive FPs can detect the pH changes in live cells, hence we can monitor the real-time progression of autophagic stages. Therefore, these unique features of FP-based autophagy biosensors and elaborate fluorescence imaging techniques can contribute to understand complex mechanisms of autophagy and the related diseases.

Most of FP-based biosensors have been developed and applied to investigate the complex mechanisms of macroautophagy. Further development and applications of FP-based biosensors targeting other types of autophagy, i.e., microautophagy and CMA, will be important to unveil the molecular mechanisms of these other types of autophagy. In addition to the mitophagy biosensor, the FP-based biosensors for other selective macroautophagy, such as ERphagy, aggrephagy, lipophagy, ribophagy or aggrephagy [161], can be further developed with specific targeting sequences to these molecules or organelles [162]. These FP-based biosensors to detect other types of autophagy will allow for the understanding of the molecular mechanisms for other autophagy types.

The FP-based biosensors are useful tools to sensitively monitor the autophagy process in live cells. It would be valuable if we could accurately control the autophagy process with high spatiotemporal resolution. For example, an optobiochemical tool to induce mitophagy has recently been reported [116]. It is composed of two parts: the first part is Venus-LID-ActA which is tethered at mitochondria via ActA, and the second part is AMBRA1-RFP-sspB which includes AMBRA1 for the induction of autophagy. Upon illumination of blue light, light-induced dimer (LID) in the first part can expose its binding site for sspB peptide [163], thus the second part, AMBRA1-RFP-sspB, can be subsequently recruited to Venus-LID-ActA located at mitochondria. The recruited AMBRA1 then successfully induces mitophagy. Further development of optobiochemical tools [164] and spatiotemporally fine control of a particular stage in different autophagy types will be useful to investigate the mechanisms of autophagy. Taken together, FP-based autophagy biosensors and novel optobiochemical tools can be applied to investigate in-depth mechanisms of autophagy and the related diseases.

## Figures and Tables

**Table 1:** Different fluorescent proteins and their physicochemical properties. Different properties of various fluorescent proteins (FPs) are summarized: maximal excitation and emission wavelengths, pKa, extinction coefficient (EC), quantum yield (QY), brightness (a relative brightness, % of enhanced green fluorescent protein (EGFP)), fluorescence fold change between pH 5.5 and 7.5.

Fluorescent Protein	λ_ex_	λ_em_	pKa	EC	QY	Brightness	Fluorescence Fold Change (pH 5.5–7.5)	Reference
mTagBFP2	399	454	2.7	50,600	0.64	32.38	1	[32], This work
mTurquoise2	434	474	3.1	30,000	0.93	27.9	-	[12,24]
CFP	456	480	-	-	-	-	-	[24]
PS-CFP2(cyan)	400	468	-	43,000	0.2	8.6	-	[24]
pHRed	440	610	6.6	-	-	-	-	[33]
mKeima	440	620	6.5	14,400	0.24	3.46	-	[23,24]
EGFP	488	507	6	55,900	0.6	33.54	1	[24], This work
PS-CFP2(green)	490	511	-	47,000	0.23	10.81	-	[24]
mWasabi	493	509	6.5	70,000	0.8	56	-	[24,28]
SE-pHluorin	495	512	7.2	-	-	-	50	[16]
pHoran1	547	564	6.7	-	-	-	10	[16]
pHoran4	547	561	7.5	-	-	-	17	[16]
mOrange	548	562	6.5	71,000	0.69	48.99	5	[16,24]
mApple	548	592	6.5	75,000	0.49	36.75	4	[16,24]
pHTomato	550	580	7.8	-	-	-	-	[14]
tdTomato	554	581	4.7	138,000	0.69	95.22	-	[24]
mNectarine	558	578	6.9	58,000	0.45	30	6	[22,24]
DsRed	558	583	-	72,500	0.68	49.3	-	[24,25]
pHmScarlet	562	585	7.4	85,000	0.47	39.73	-	[26]
PAmCherry1	564	595	6.3	18,000	0.46	8.28	-	[24,31]
pHuji	566	598	7.7	31,000	0.22	6.82	22	[16,24]
mScarlet	569	594	5.3	100,000	0.7	70	1.3	[24], This work
mRFP1	584	607	4.5	50,000	0.25	12.5	-	[14,21]
mCherry	587	610	4.5	72,000	0.22	15.84	1	[25], This work
mKate2	588	633	5.4	62,500	0.4	25	1.3	[23], This work
mKate	588	635	6.2	45,000	0.33	14.85	-	[24,29]
HcRed	592	645	-	20,000	0.015	0.3	-	[24]

The colors represent the colors for particular wavelengths of light.

**Table 2:** FP-based autophagy biosensors and their applications to study neurodegenerative diseases.

FP-Based Autophagy Biosensor type	Molecular Design	Detected Autophagy Types (and Stages)	Biosensor Applications in Neurodegenerative Disease
FP-LC3	EGFP-LC3 RFP-LC3 mCherry-LC3 YFP-LC3 HcRed-LC3 CFP-LC3	Macroautophagy (autophagosome)	Huntingtin disease [68] Alzheimer’s disease [71,146,147,148,149] Parkinson’s disease [150,151,152,153]
HyD-LIR-GFP	HyD-LIR(Fy)-GFP HyD-LIR(TP)-GFP	Macroautophagy (autophagosome)	-
RFP-GFP-LC3	mRFP-GFP-LC3 mCherry-EGFP-LC3 mTagRFP-mWasabi-LC3 mKate-SEP-LC3	Macroautophagy (autophagosome, autolysosome)	Huntingtin disease [154,155,156] Alzheimer’s disease [73,157,158] Parkinson’s disease [159,160]
GFP-LC3-RFP-LC3ΔG	GFP-LC3-RFP-LC3ΔG	Macroautophagy (autophagosome, autolysosome)	-
BFP-RFP-GFP-LC3	mTagBFP2-mApple-SEP-LC3	Macroautophagy (phagophore, autophagosome, fusion, autolysosome)	Alzheimer’s disease [73]
pH-Lemon-LC3	mTurquioise2- EYFP-LC3	Macroautophagy (autophagosome, autolysosome)	-
mito-SRAI	mito-TOLLES-Ypet	Mitophagy (autophagosome, autolysosome)	-
CMA sensor	KFERQ-PS-CFP2 KFERQ-PA-mCherry	Chaperon-mediated autophagy	-

## Abbreviations

Aβ	amyloid-beta
AD	Alzheimer’s disease
AMBRA1	activating molecule in Beclin 1-regulated autophagy protein 1
AMPK	AMP-activated protein kinase
Atg	autophagy-related protein
BFP	blue fluorescent protein
CMA	chaperone-mediated autophagy
ECFP	enhanced cyan fluorescent proteins
EYFP	enhanced yellow fluorescent protein
FIP200	focal adhesion kinase family interacting protein of 200-kDa
FP	fluorescent protein
FRET	fluorescence resonance energy transfer
GFP	green fluorescent protein
HD	Huntington’s Disease
Hsc70	heat shock cognate protein of 70 kDa
Htt	huntingtin
HyD	hydrophobic domain
LAMP2A	lysosomal-associated membrane protein 2A
LC3	microtubule-associated protein light chain 3
LDH	lactate dehydrogenase
LD	lipid droplet
LID	light-induced dimer
LIR	LC3 interacting region
mTOR	*mechanistic target of rapamycin*
NBR1	neighbor of BRCA1 gene 1
NDP52	nuclear dot protein 52 kDa
PD	Parkinson’s disease
PE	phosphatidylethanolamine
PINK1	PTEN-induced kinase 1
PI3P	phosphatidylinositol 3-phosphate
PolyQ	polyglutamine
PS-CFP2	photoconvertible cyan FP
PS-1	presenilin 1
RFP	red fluorescent protein
SEP	superecliptic pHluourin
SF44	Seoul-Fluor 44
SILAC	stable isotope labeling by amino acid in cell culture
SNARE	soluble NSF (N-Ethylmaleimide-Sensitive Factor) Attachment Protein Receptor
SRAI	signal-retaining autophagy indicator
TEM	transmission electron microscope
TOLLES	TOLerance of Lysosomal EnvironmentS
ULK1	Unc-51 like kinase 1
Vps34	vacuolar protein sorting 34
WIPI	WD-repeat protein interacting with phosphoinositide
